# Pathological and Functional Brain Amyloids: A New Concept Explaining the Differences

**DOI:** 10.3390/ijms262110459

**Published:** 2025-10-28

**Authors:** Alexey P. Galkin, Vladimir A. Mitkevich, Alexander A. Makarov, Anna A. Valina, Evgeniy I. Sysoev

**Affiliations:** 1 St. Petersburg Branch, Vavilov Institute of General Genetics, Russian Academy of Sciences, 199034 St. Petersburg, Russia; anna.valina@spbu.ru (A.A.V.); st063251@student.spbu.ru (E.I.S.); 2 Department of Genetics and Biotechnology, Faculty of Biology, St. Petersburg State University, 199034 St. Petersburg, Russia; 3 Engelhardt Institute of Molecular Biology, Russian Academy of Sciences, 119991 Moscow, Russia; mitkevich@eimb.ru (V.A.M.); aamakarov@eimb.ru (A.A.M.)

**Keywords:** functional vs. pathological amyloids, brain, neurodegenerative diseases, comparative analysis, amyloidogenic cores, neurotoxicity, amyloids’ functional partners, cellular localization, pathological targets, protein misfolding

## Abstract

In recent years, amyloid proteins that perform vital functions in the brain have been characterized. The question of why some amyloids are neurotoxic while others are harmless remains open. Here, we provide a brief overview of pathological and functional brain amyloids and present a comparative analysis of their amino acid sequences based on the percentage of hydrophobic and charged residues, as well as their enrichment in glutamine, asparagine, serine, and glycine. We demonstrate that pathological and functional brain amyloid proteins, along with their amyloidogenic fragments, do not differ in amino acid composition, contrary to previous assumptions. The ability of an amyloid to cause toxicity can instead be explained by the concept of “available targets”. Evidence from studies of pathological amyloids demonstrate that their toxicity is determined not only by a loss of function but also by aberrant interactions with specific targets, such as PrP^C^ or mitochondrial membranes. Binding to these targets triggers pathological cascades that ultimately lead to cell death. In contrast, such targets are inaccessible to functional amyloids, either because of localized translation and protein sequestration within specialized cellular structures, or because their interactions with physiological partners prevent binding to dangerous targets.

## 1. Introduction

Amyloids are protein fibrils with a cross-β structure. While intramolecular β-sheets are common structural motifs in proteins, the formation of intermolecular cross-β structures is a rare event that leads to protein aggregation. Often, the appearance of amyloid oligomers and fibrils is associated with incurable diseases known as amyloidoses. Pathological amyloids formed by proteins such as PrP, Aβ, tau, α-synuclein, TDP-43, and mutant huntingtin are cytotoxic and cause neuronal cell death [1]. However, the etiology of neurodegenerative diseases is complex, and amyloid formation is not always the primary cause. Accordingly, pathologies such as Alzheimer’s, Parkinson’s, and Huntington’s diseases, as well as tauopathies and amyotrophic lateral sclerosis, are not typically classified as amyloidoses.

Until the early 21st century, amyloid fibrils were considered exclusively pathological. However, over the past quarter-century, numerous proteins have been described that function normally in the amyloid form. These functional amyloids have been discovered in bacteria, fungi, plants, and animals, including humans [2,3,4,5,6,7,8,9,10,11,12]. Amyloids represent a special form of protein folding, and it is not surprising that, during evolution, some proteins have acquired the ability to form cross-β fibrils that provide adaptive advantages. Thus, the view of amyloids as exclusively cytotoxic structures is now considered outdated. Several proteins that are stored or function in the amyloid form are present in the brain. For example, the amyloid form of the FXR1 protein binds RNA [7], while amyloid fibrils of MBP stabilize the myelin structure [10] in the vertebrate brain. Amyloid oligomers of the Orb2 protein regulate translation in the fruit fly brain neurons [8]. Furthermore, several mammalian peptide and protein hormones are stored in amyloid form within the secretory granules of the pituitary gland [11].

The question of why some amyloids are cytotoxic to neuronal cells while others are not remains unresolved. In this review, we perform a comparative analysis of functional and pathological brain amyloids and discuss the factors that determine the cytotoxicity of the latter. We focus only on brain proteins whose amyloid properties have been clearly established. Over the past two years, advances in cryo-electron microscopy have enabled the structural characterization of several pathological amyloids associated with familial forms of amyotrophic lateral sclerosis (ALS) and frontotemporal lobar degeneration (FTLD) [13,14]. However, the molecular mechanisms underlying the pathogenesis of these amyloids have not yet been elucidated. Therefore, we did not include these amyloids in our comparative analysis. Some studies have shown that a protein or its fragment can form amyloid fibrils in vitro or when overproduced in a heterologous system. This alone does not prove that the protein forms such fibrils under physiological conditions. In recent decades, there has been a growing tendency to label proteins as “amyloid-like” if they share sequence similarity with known amyloids or have been characterized only in vitro or in heterologous systems. While such data may be useful for identifying potential amyloids, they are not considered in this review. In this work, we compared functional and pathological amyloids based on parameters such as amino acid composition, localization, and their interaction with various molecular targets. A comparative analysis of brain proteins with well-established amyloid properties allows us to propose a concept explaining why some amyloids are toxic to brain cells while others are not.

## 2. Functional Amyloids

### 2.1. Myelin Basic Protein

Myelin basic protein (MBP) is a key structural and functional component of the central nervous system (CNS) myelin, constituting nearly one-third of its total protein mass [15]. It is encoded by the *Golli/Mbp* locus, which, through alternative splicing, produces both classic MBP isoforms, restricted to myelinating oligodendrocytes, and Golli proteins, which are more broadly expressed [16,17]. The mRNA encoding classical MBP isoforms is transported to the oligodendrocyte processes, where local translation occurs [18]. Translation is actively repressed during transport to prevent premature MBP accumulation [19,20]. This spatial regulation ensures that MBP is produced exactly at sites where the new myelin membrane needs to be compacted. The N- and C-termini of MBP molecules bind to lipids on opposite membranes in the flattened oligodendrocyte processes [21]. This event is accompanied by the displacement of other proteins located in this region [22]. Following membrane attachment, MBP becomes compacted, resulting in the formation of an electron-dense line 3 nm thick between the membranes [23].

**Figure 1:**
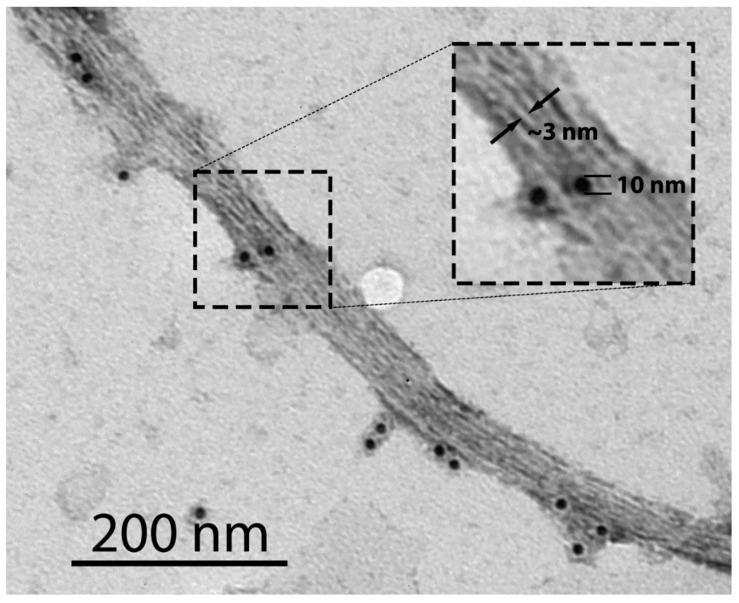
TEM image of MBP amyloid fibrils immunoprecipitated from the brain of the rat *Ratus norvegicus*. PAA539Mi01 was used as a primary rabbit anti-MBP antibody. A goat anti-rabbit gold-conjugated antibody with a gold particle (10 nm) was used as a secondary antibody. Immunoprecipitation was performed as described previously [10]. The inset shows a magnified region of the image, highlighting individual protofibrils with a thickness of ~3 nm (indicated by arrows).

It was previously shown that the corpus callosum in wild-type mice was stained with the amyloid-specific dye Thioflavin S (ThS), whereas the corresponding brain region in mice with MBP deletion was not [22]. Another study found that MBP is present in the rat brain in the form of SDS-resistant amyloid-like aggregates [7]. Recently, we obtained direct evidence that MBP is present in the vertebrate brain in amyloid form [10]. This protein clearly colocalizes with Congo Red (CR) and ThS on brain sections of the chicken *Gallus gallus domesticus*, the common frog *Rana temporaria*, the red-eared slider *Trachemys scripta*, and the rat *Rattus norvegicus*. Purified MBP fibrils obtained from brain tissue are detectable by transmission electron microscopy (TEM) and exhibit apple-green birefringence after CR staining [10]. In Figure 1, protofibrils of immunoprecipitated MBP labeled with gold are shown; their thickness is ~3 nm, indicating that the main dense line between the oligodendrocyte membranes corresponds to MBP amyloid protofibrils.

Importantly, the central domain of MBP (residues 60–119) is essential for amyloid formation: this region confers aggregation capacity both in a yeast model system and in a cell-free system, where it forms twisted amyloid fibrils. Based on these findings, we proposed a model in which MBP not only bridges opposing cytoplasmic membranes via its N- and C-termini but also forms longitudinal “amyloid stitches” that reinforce the multilamellar myelin sheath, thereby providing mechanical stability to compact myelin [10].

While MBP is protective within compact myelin, mislocalized MBP can become neurotoxic. Experimental studies have shown that extracellular MBP binds to neuronal plasma membranes, depolarizes them, increases Ca^2+^ influx, and ultimately induces neuronal death [24]. In lipid vesicle assays, MBP disrupted bilayer integrity, suggesting a direct membrane-permeabilizing mechanism [24]. These findings indicate that MBP, once displaced from its physiological context, can act as a membrane-active toxin. Also, it has been shown that in Alzheimer’s disease (AD), against the background of neuronal death and myelin sheath destruction, MBP is present within extracellular Aβ plaques [25].

### 2.2. FXR1 and Orb2

The FMR1 autosomal homolog 1 (FXR1) is an RNA-binding protein that belongs to the Fragile X-related (FXR) family [26]. Although *FXR1* is broadly expressed across different tissues, the highest levels are found in muscle and brain, particularly in neurons and Purkinje cells [27]. Within cells, FXR1 has predominantly cytoplasmic localization, where it forms ribonucleoprotein (RNP) particles. The FXR1 protein is critical for development and survival, as *FXR1* knockout mice die at the neonatal stage, most likely due to cardiac or respiratory failure resulting from impaired mRNA transport and translational control in muscle [28]. Conditional *FXR1* knockout in the excitatory forebrain neurons of mice demonstrated selective enhancement of long-term spatial memory storage, hippocampal long-lasting synaptic plasticity, and de novo GluA2 synthesis [29].

Under normal conditions, FXR1 is associated with translational silencing through binding to AU-rich elements (AREs) located in the 3′ untranslated regions (UTRs) of many oncogenes, cytokine, and growth factor mRNAs [30]. FXR1 has been identified as a negative regulator of mRNAs encoding inflammatory proteins, including TNFα, ICAM-1, IL-1β, and MCP-1 [31]. This protein may also stabilize and activate translation of its targets. For example, it binds to AREs within *cMYC* mRNA, enhances transcript stability, and promotes the recruitment of the eIF4F complex to translation initiation sites, thereby facilitating *cMYC* translation [32].

The RNA-binding activity of FXR1 is mediated by three arginine-rich motifs (RG, RGG, and R) that interact with nucleic acid structures such as G-quadruplexes [33], as well as by two KH domains that bind RNAs containing pseudoknots or kissing loops [34]. In addition to RNA-binding domains, FXR1 harbors multiple protein–protein interaction domains. These include the KH domains and RG/RGG motifs, which are multifunctional and bind both RNA and proteins, as well as two Tudor domains, a KH0 domain, and coiled-coil regions [35]. Tudor domains mediate dimerization and recognize methylated arginines [36,37]. Since the KH0 domain lacks the GXXG motif required for RNA binding, it likely functions exclusively in protein–protein interactions [38]. Beyond its homologues, FXR1 has been shown to interact with a wide range of partners, including cytoskeletal proteins such as Arp2 and CYFIP1 [39], the Argonaute 2 protein involved in microRNA-mediated decay [40], as well as many other protein partners [41].

FXR1 has previously been shown to function in amyloid form in the cytoplasm of cortical neurons across multiple vertebrate species [7,42]. Immunohistochemical assays of brain slices revealed clear colocalization of FXR1 with amyloid-specific dyes CR and ThS in the neuronal cytoplasm. FXR1 fibrils immunoprecipitated from brain lysates exhibit characteristic yellow-green birefringence after CR staining. The amyloidogenic region of FXR1 corresponds to its N-terminal fragment (residues 1–379) [7].

**Figure 2:**
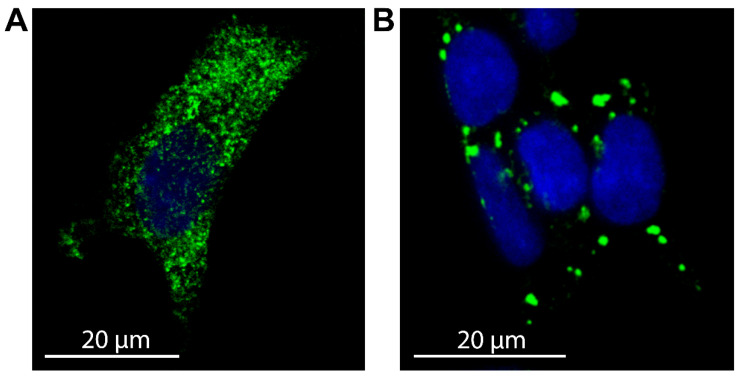
FXR1 localization in SH-SY5Y cells under physiological and stress conditions. (**A**) FXR1 forms cytoplasmic oligomers (small grains) in the human neuroblastoma SH-SY5Y cell line under physiological conditions. (**B**) FXR1 is recruited into large stress granules upon treatment of cells with 3 mM sodium arsenite for 1 h. Rabbit anti-FXR1 antibodies DF12402 was used as the primary antibody, and goat anti-rabbit IgG(H+L) antibody conjugated with Alexa Fluor 488 as the secondary (green fluorescence). Nuclei were stained with Hoechst 33342 (blue fluorescence). The immunocytochemistry assay was performed as described previously [43].

FXR1-containing RNP particles were shown to stabilize associated mRNAs, as transcripts colocalized with FXR1 were resistant to high concentrations of RNase A [7]. Experiments using the human neuroblastoma cell line SH-SY5Y provided higher-resolution insights into the subcellular localization and aggregation level of FXR1 [43]. Normally, FXR1 forms small cytoplasmic oligomers or grains (Figure 2A). These observations suggest that interactions with RNA molecules and RNA-binding proteins limit the growth of FXR1 amyloid fibrils. Upon oxidative stress or heat shock, small FXR1-containing particles are recruited into stress granules—transient, membraneless RNP complexes that assemble through protein/RNA phase separation and promote cell survival (Figure 2B). Importantly, the amyloid properties of FXR1 were shown to be preserved during its incorporation into stress granules [43]. However, FXR1 recruitment into stress granules appears to occur through non-amyloid protein–protein interactions, consistent with the highly dynamic and reversible nature of these structures that disassemble upon stress resolution.

The *Drosophila melanogaster* protein Orb2, like vertebrate FXR1, is an RNA-binding protein that regulates translation [44]. Orb2 is a member of the cytoplasmic polyadenylation element-binding (CPEB) family. This evolutionarily conserved group of RNA-binding proteins regulates mRNA transport and local translation, processes essential for early embryonic development, synaptic plasticity, and long-term memory [45,46]. While mammalian CPEB protein fragments have been shown to form amyloid fibrils in vitro, clear evidence for amyloid properties in the brain has been demonstrated conclusively only for the fruit fly Orb2 protein [8]. The authors of that study isolated Orb2 from approximately three million fruit fly heads and determined the structure of its native fibrils using cryo-electron microscopy. The amyloidogenic core of Orb2 has been shown to comprise amino acid residues 176 to 206 and to be enriched in glutamine residues. Orb2 binds to the UTRs of various target mRNAs via its RNA recognition motifs to regulate their translation [47]. The monomeric form of Orb2 represses translation and shortens mRNA poly(A) tails, whereas the amyloid oligomeric form enhances translation and elongates poly(A) tails [44]. Orb2 has also been shown to interact with several proteins involved in translation initiation, mRNA binding, and synaptic activity [48].

### 2.3. Peptide and Protein Hormones in Secretory Granules

Neuroendocrine cells, as well as peptidergic neurons, are known to form large vesicles with electron-dense cores, commonly referred to as secretory granules. These membrane-coated granules serve as inert storage sites for specific secretory proteins and peptides over extended periods, allowing the cell to release them rapidly and massively by regulated exocytosis [49,50]. Growth hormone, prolactin, adrenocorticotropic hormone (ACTH), β-endorphin, oxytocin, and vasopressin have been shown to form storage granules in the pituitary gland [51,52,53,54]. Proteins and peptides concentrated in secretory granules exist as reversible aggregates formed by self-association [55]. The degree of concentration is remarkable: in the case of prolactin, its level in granules is approximately 200 times higher than in the endoplasmic reticulum [56]. Upon stimulation of neuroendocrine cells, the granule content is released in soluble form into the extracellular space [49].

The aggregation state of proteins in secretory granules ranges from amorphous to crystalline form, ensuring their stable and dense packaging for regulated secretion [49,50]. Direct evidence for the presence of amyloid material in the secretory granules of pituitary neuroendocrine cells has been obtained. Maji and colleagues purified secretory granules from the mouse pituitary tumor neuroendocrine cell line AtT20 and analyzed their amyloid properties ex vivo [11]. AtT20 cells are known to synthesize, correctly glycosylate, and process precursors of ACTH and β-endorphin, thereby producing the mature forms of these hormones and storing them properly in secretory granules [57].

It was shown that purified protein contents from AtT20 secretory granules bind conformation-dependent amyloid-specific antibodies OC, whereas monomeric ACTH and β-endorphin do not. Moreover, purified granules from AtT20 cells were demonstrated to bind Thioflavin T, CR, and exhibit birefringence under cross-polarized light upon CR binding. Finally, X-ray fiber diffraction of purified membraneless secretory granules provided strong evidence for amyloid fibrils. A preparation of granules without membrane was employed, since membrane lipids produce a pronounced reflection at 4.1 Å, which is close to the 4.7 Å reflection characteristic of a cross-β-sheet structure. Thus, it can be concluded that at least ACTH and β-endorphin included in secretory granules are of amyloid nature. Similar results were obtained by analysis of secretory granules purified from rat pituitary using the same methods. Immunohistochemical studies of colocalization of hormone-specific antibodies with ThS or OC antibodies on mouse pituitary cryosections confirmed that neuroendocrine hormones such as ACTH, β-endorphin, prolactin, growth hormone, oxytocin, and vasopressin display amyloid properties [11]. However, it is worth noting that these molecules perform their physiological functions in the monomeric form, whereas the amyloid conformation serves exclusively for storage.

Protein/peptide hormones do not exhibit toxicity because they lack the ability to interact with other proteins within the densely packed, membrane-enclosed secretory granule. Amyloid aggregation of these hormones during secretory granule formation is highly regulated by processing events and seeding requirements, including high local concentration, acidic pH, elevated Ca^2+^ levels, and helper molecules such as granins or glycosaminoglycans. This aggregation is strongly localized to the trans-Golgi network, preventing cytotoxic effects [55,58,59]. Disruption of localized aggregation or improper sorting, however, can give rise to cytotoxicity. For instance, in diabetes insipidus, folding-deficient mutants of provasopressin are retained in the endoplasmic reticulum and produce cytotoxic fibrillar aggregates [60].

## 3. Pathological Amyloids

### 3.1. Prion Protein

The cellular prion protein (PrP^C^) is anchored to the neuronal membrane via glycosylphosphatidylinositol and can act as a receptor or transducer of extracellular signals [61,62,63]. In neurons, PrP^C^ is predominantly localized in the pre- and postsynaptic compartments of nerve terminals. It binds divalent cations such as copper and zinc and interacts with various cellular receptors, including the NMDA receptor [64,65]. PrP^C^ is involved in regulating neuritogenesis, neuronal homeostasis, cell signaling, cell adhesion, and stress responses [66].

The pathological amyloid form of PrP is designated Prion Protein Scrapie (PrP^Sc^) [67]. The formation of PrP^Sc^ fibrils occurs sporadically or results from aggregation-promoting mutations. PrP^Sc^ particles are infectious and can be transmitted through contact with infected animals or consumption of contaminated feed. These fibrils exhibit remarkable resistance to various treatments, including boiling, certain autoclaving conditions, and degradation by gastrointestinal proteases [67,68]. Following ingestion, PrP^Sc^ particles from the gastrointestinal tract enter the lymphatic system and penetrate follicular dendritic cells (FDCs), which produce their own PrP^C^. Attachment of the exogenous PrP^Sc^ particles to endogenous PrP^C^ induces conformational conversion of the latter into the amyloid form [69]. Although PrP^Sc^ replicates within FDCs, it does not cause toxicity. As FDCs mature, they migrate to the spleen, where they contact cells of the peripheral nervous system [70]. Subsequently, PrP^Sc^ particles are internalized by cells of the peripheral nervous system and later spread to the central nervous system. Through intracellular transport, PrP^Sc^ reaches the brain, bypassing the blood–brain barrier (BBB), and is released into the extracellular space.

Prion infections can undergo interspecies transmission. For instance, prion disease can be contracted by consuming contaminated meat, as seen in variant Creutzfeldt-Jakob Disease (vCJD) resulting from bovine spongiform encephalopathy (BSE, or “mad cow disease”) [71]. Cattle, in turn, became infected through feed containing meat and bone meal derived from sheep affected by scrapie [72]. However, prion infection is not transmitted directly from infected sheep to humans [73]. This phenomenon is known as the “species barrier” for prion transmission. The presence or absence of this barrier is determined by similarities and differences in the PrP sequences among mammalian species. Variations in PrP amino acid sequences can prevent the conversion of PrP^C^ to its pathological counterpart, PrP^Sc^, when infectious prion particles from another species enter an organism. PrP^Sc^ particles arising sporadically, through mutation, or via infectious transmission, cause various forms of incurable prion diseases in humans and animals, all of which lead to neurodegeneration and fatal outcomes [74]. These forms differ in the brain regions predominantly affected, incubation periods, and clinical symptoms.

Experimental data indicate that PrP^Sc^ oligomers exhibit the highest toxicity [75]. Current understanding of PrP^Sc^ toxicity mechanisms is discussed in a recent review [65]. PrP^Sc^ interacts with the C-terminal domain of PrP^C^, inducing its conversion into the amyloid form [76]. This interaction liberates the N-terminal domain, enabling it to initiate toxic activity at the cell surface [77]. The conversion of PrP^C^ to PrP^Sc^ triggers the opening of NMDA receptors, leading to an influx of Ca^2+^. This event activates the MAPK p38 pathway and its downstream effectors MK2/3, ultimately resulting in the collapse of the actin cytoskeleton and dendritic spines [78]. Furthermore, this conversion triggers translation repression via activation of the unfolded protein response, mediated by PERK-dependent phosphorylation of eukaryotic initiation factor 2α [79]. Importantly, PERK inhibition prevented neuronal death and behavioral symptoms despite ongoing PrP^Sc^ accumulation [80]. Scrapie infection also induces mitochondrial reactive oxygen species (mtROS) production and loss of mitochondrial membrane potential. These events promote abnormal mitochondrial fission and mitophagy, leading to caspase-3 activation and apoptosis [81]. The authors attribute apoptosis initiation to altered mitochondrial Ca^2+^ concentration and increased mtROS, concurrent with the conversion of PrP^C^ to PrP^Sc^ on the cell plasma membrane.

Prion infection additionally activates microglia and astrocytes [82,83,84]. In the early stages, this activation can transiently delay PrP^Sc^ proliferation and spread, but prolonged activation leads to chronic neuroinflammation. In advanced disease, high levels of pro-inflammatory cytokines and persistent astrocyte activation contribute to synaptic disruption and neurodegeneration [85]. Despite the diversity of downstream mechanisms, all prion-induced neurotoxic pathways are associated with direct or indirect interactions of the prion protein with targets such as cellular receptors, microglia, and astrocytes.

### 3.2. Aβ Peptide

Amyloid-β (Aβ) peptide oligomers are the primary cytotoxic factor in AD. Although the etiology of AD is complex, the formation of extracellular Aβ amyloid conformers and intracellular aggregates of hyperphosphorylated tau protein is a defining pathological hallmark. AD is the most common neurodegenerative disorder, with a strong correlation between incidence and age. Currently, over 50 million people worldwide are affected by AD [86]. Most cases of AD are sporadic, whereas less than 5% exhibit a hereditary predisposition, most often associated with mutations in the *APP*, *PSEN1/2*, and *APOE* genes [87]. These mutations directly or indirectly promote Aβ peptide accumulation in the brain. Aβ is generated by proteolytic processing of the transmembrane amyloid precursor protein (APP) by β- and γ-secretases [88]. Numerous Aβ species exist, with Aβ40 being the most abundant (80–90%) and Aβ42 accounting for 5–10%. Aβ production is a normal physiological process that does not inherently cause pathology. Aβ acts as a modulator of synaptic activity and a component of the brain’s innate immunity by regulating neurotransmitter release and neuronal receptor function [89,90,91]. It also possesses antimicrobial properties by binding to and neutralizing pathogens in the brain [92].

The excessive accumulation of the Aβ peptide in the extracellular space can induce the formation of cytotoxic amyloid conformers. Among them, Aβ42 oligomers demonstrate the highest toxicity [93]. The accumulation of neurotoxic Aβ oligomers (AβOs) clearly underlies hereditary AD forms, whereas the causes of sporadic AD remain unclear. In sporadic cases, the formation of neurotoxic Aβ likely represents one stage of a pathological cascade. Besides age, risk factors for sporadic AD include brain trauma, vascular disorders, metabolic syndromes, and aluminum accumulation [87,94]. However, only a small percentage of people under 65 suffer from AD, whereas its prevalence among individuals aged ≥95 in the USA reaches about 50% [95]. Given this strict age dependence, AD onset may be linked to changes in the level of an uncharacterized aging-dependent hormonal factor responsible for Aβ clearance. Aβ clearance occurs through both enzymatic and non-enzymatic pathways [96]. Key non-enzymatic clearance is mediated by the glymphatic system, which eliminates Aβ during sleep [97], as well as by transport across the BBB via LRP1 (efflux) and RAGE (influx) receptors [98]. Enzymatic clearance depends on Aβ degradation by enzymes such as neprilysin (NEP) and insulin-degrading enzyme (IDE) [99]. Impairment of any of these mechanisms promotes Aβ accumulation in the brain.

The toxicity of AβOs primarily involves mitochondrial damage, impairment of the endosome-lysosome system, cellular membrane disruption, hyperphosphorylation and polymerization of tau protein into neurofibrillary tangles, ultimately leading to neuronal death. Aβ amyloid conformers interact with the N-terminal domain of PrP^C^ on cortical neuron membranes [100]. This interaction induces tau hyperphosphorylation, resulting in synaptic loss [101]. Additionally, this interaction activates Fyn kinase, which phosphorylates the NR2B subunit of the NMDA receptor, transiently elevating surface NR2B levels and leading to excitotoxicity and destabilization of dendritic spines [102]. Some evidence suggests that AβOs directly interact with NMDA receptors containing GluN2B subunits and with metabotropic glutamate receptor 1 in primary cortical neurons [103]. Binding to these receptors likely induces various pathological responses that impair synaptic function. Aβ42 oligomers can also insert into cellular membranes, forming large ion-channel pores that disrupt intracellular Ca^2+^ homeostasis [104]. Mitochondria represent another direct target of AβOs [105]. AβOs can be internalized and imported into mitochondria via the translocase of the outer membrane (TOM) complex, accumulating within mitochondrial cristae [106]. This interaction disrupts mitochondrial morphology, interferes with electron transport chain processes, induces oxidative stress, and impairs mitochondrial fission-fusion dynamics, ultimately disrupting mitochondrial function [107]. Similarly to PrP^Sc^, AβOs accumulation activates microglia and astrocytes, which attempt to clear the toxic conformers [108]. However, this activation becomes chronic in AD, accelerating neurodegeneration. In conclusion, amyloid oligomers bind to multiple targets, triggering various cytotoxic cascades.

### 3.3. Tau

Tau is a microtubule-associated protein highly expressed in neurons, where it plays a critical role in stabilizing microtubules [109], regulating neurite outgrowth [110], and supporting the transport of organelles and vesicles [111]. Beyond its structural role, tau is involved in neuronal development, dendritic and synaptic plasticity, and modulation of intracellular signaling pathways [112]. Under pathological conditions, however, tau undergoes conformational changes and self-assembles into amyloid fibrils [113], which accumulate in neurofibrillary tangles (NFTs), a defining feature of AD and some other disorders. The amyloid structure of hyperphosphorylated tau filaments isolated from the brain was confirmed using cryo-electron microscopy [113]. Notably, accumulation of Aβ, particularly in its oligomeric form, has been shown to promote tau hyperphosphorylation and aggregation, acting upstream in the pathological cascade of AD [114,115].

Tau pathology is central to a group of neurodegenerative diseases collectively known as tauopathies. These include AD, progressive supranuclear palsy (PSP), corticobasal degeneration (CBD), and Pick’s disease, among others [116]. Clinically, tauopathies present with diverse phenotypes depending on the distribution and extent of tau pathology. AD primarily manifests as progressive memory impairment and difficulties with higher-order cognitive functions [116]. PSP and CBD are characterized by Parkinsonian features, including bradykinesia and rigidity [116]. Pick’s disease presents as frontotemporal dementia, with profound changes in personality, language, and behavior [116].

Post-translational modifications play key roles in tau toxicity. Hyperphosphorylation disrupts microtubule binding and promotes tau aggregation [117], while acetylation at Lys174 delays protein turnover and exacerbates accumulation [118]. Proteolytic processing generates truncated tau fragments that further enhance aggregation propensity and toxicity [119]. Notably, full-length tau in amyloid form can activate calpain-2, leading to degradation of the nicotinic acetylcholine receptor subunit 4 and disruption of cholinergic signaling, thereby creating a feedback loop that promotes tau fragmentation [120]. Pathological tau also perturbs neuronal physiology by altering calcium homeostasis, inducing dendritic spine loss, impairing axonal transport, and disrupting mitochondrial dynamics [121]. Soluble tau oligomers are particularly neurotoxic: they act as seeds for misfolding, impair synaptic plasticity, and cause mitochondrial dysfunction and memory impairment in animal models [122,123]. Phosphorylated tau interacts with mitochondria, impairing their function and contributing to neuronal toxicity [124,125]. This interaction leads to mitochondrial membrane depolarization and increased production of reactive oxygen species, thereby exacerbating cellular stress and neuronal damage [126]. Mislocalization of tau to dendrites contributes to AMPA receptor downregulation, further exacerbating synaptic failure [127]. Tau is also found in the neuronal nucleus, where it binds DNA and associates with nuclear lamina components such as Lamin B1, contributing to chromatin remodeling and nuclear envelope deformation. These nuclear alterations have been linked to neuronal vulnerability in tauopathies [128].

Tau pathology affects not only neurons but also glial cells. Microglia can internalize extracellular tau and contribute to its propagation by releasing tau-containing vesicles [129], while also promoting neuroinflammation through activation of the NLRP3 inflammasome [115]. Astrocytes can likewise take up extracellular tau [130], and its accumulation has been shown to impair astrocytic support of synapses and to exacerbate neuronal dysfunction [131]. In neuronal cell models, tau oligomers were shown to bind to PrP^C^ and muscarinic receptors, disturbing intracellular Ca^2+^ signaling [132]. Importantly, data suggest that it is the dephosphorylated, rather than hyperphosphorylated, form of extracellular tau that acts as an agonist of muscarinic M1 and M3 receptors. This interaction, mediated by tissue-nonspecific alkaline phosphatase (TNAP), provokes sustained intracellular calcium elevation and ultimately leads to neuronal death [133]. It has further been demonstrated that extracellular tau, particularly when associated with extracellular vesicles isolated from AD brains, is efficiently taken up by neurons, where it seeds aggregation of endogenous tau and propagates tau pathology in vivo [134]. This prion-like mechanism is thought to underlie the stereotypical spread of tau pathology across interconnected brain regions in tauopathies.

In summary, tau is a physiologically essential protein that maintains neuronal structure and function. However, under pathological conditions, it adopts amyloid conformations that drive neurodegeneration. Misfolded or extracellular tau becomes toxic when it interacts with vulnerable cellular components, including receptors, organelles, and signaling pathways, leading to calcium dysregulation, synaptic failure, and neuronal death.

### 3.4. α-Synuclein

α-Synuclein (α-syn) is a small, intrinsically disordered protein enriched at presynaptic terminals, where it regulates synaptic vesicle trafficking and neurotransmitter release. Under pathological conditions, α-syn misfolds into cross-β structures ranging from soluble oligomers to fibrils [135]. These aggregates accumulate in Lewy bodies and Lewy neurites, which are the histopathological hallmarks of Parkinson’s disease (PD) and related synucleinopathies. In these disease contexts, α-syn behaves as a pathological amyloid, acquiring conformations that confer cytotoxic properties [136]. Most cases of PD are sporadic, typically occurring in people over the age of 60. The disease can be triggered by various risk factors, including brain injuries, exposure to environmental toxins, and oxidative stress, which promote α-syn misfolding and the formation of toxic oligomers [137,138]. Amyloid conformers of α-syn contribute to the degeneration of dopaminergic neurons in the substantia nigra, leading to the characteristic motor and cognitive symptoms of PD.

A central debate concerns which α-syn species are most toxic. Although inclusions are a defining neuropathological feature, evidence from post-mortem human tissue and model systems suggests that soluble oligomeric species, rather than mature fibrils, are primarily responsible for cytotoxicity [139]. Different oligomeric “strains” may also account for the heterogeneity of clinical phenotypes across synucleinopathies [140].

At the synapse, pathological α-syn perturbs vesicle trafficking and SNARE-complex function. Aggregated α-syn binds synaptobrevin-2, blocking SNARE complex assembly and vesicle docking [141], while clusters of oligomer-bound vesicles impair recycling and neurotransmitter release [142]. α-syn in amyloid form also interferes with dopaminergic neurotransmission by inhibiting tyrosine hydroxylase, the rate-limiting enzyme in dopamine biosynthesis [143], and by binding the dopamine transporter, thereby reducing dopamine reuptake [144]. Moreover, α-syn oligomers can insert into vesicle membranes and form pore-like structures, causing neurotransmitter leakage, which in the case of dopaminergic neurons exacerbates oxidative stress [145].

Mitochondrial dysfunction represents another critical pathway of α-syn toxicity. α-syn has been identified within mitochondria in patient tissue and transgenic models [146]. Its association with mitochondrial membranes disrupts organellar architecture, reduces complex I activity, and promotes abnormal fragmentation [139]. These changes impair ROS handling and can lead to mtDNA damage and defective mitophagy. Notably, *SNCA* knockout mice exhibit reduced ROS production and resistance to mitochondrial toxins [147], underscoring the role of α-syn in oxidative stress.

Pathological interactions with the cytoskeleton and nucleus further broaden α-syn’s toxic profile. α-syn binds α- and β-tubulin, co-aggregating with tubulin and impairing microtubule polymerization, vesicle trafficking, and neurite integrity [148]. Within the nucleus, α-syn binds histones and DNA, inhibiting histone acetylation and impairing DNA repair [149]. These interactions may underlie transcriptional dysregulation observed in synucleinopathies.

Beyond neurons, α-syn toxicity involves glial activation, contributing to neuroinflammation. Pathological α-syn species activate microglia, promoting the release of pro-inflammatory cytokines and exosomes that can facilitate prion-like α-syn propagation and exacerbate dopaminergic neuron loss [150,151]. The microglial responses vary depending on activation state and α-syn burden, with overloaded or pro-inflammatory microglia amplifying oligomer transmission, while resting microglia can partially restrain it [150,152]. Similarly, astrocytes internalize oligomeric and fibrillar α-syn, leading to mitochondrial dysfunction, oxidative stress, and pro-inflammatory cytokine production [153]. α-syn activated astrocytes reduce their trophic support for neurons, which contribute to neuronal death [153]. Together, these glial responses amplify neurotoxicity and promote disease progression.

These findings demonstrate that α-syn toxicity is multifaceted, spanning synaptic dysfunction, membrane permeabilization, mitochondrial stress, cytoskeletal disruption, and nuclear dysregulation. These harmful effects occur when α-syn, normally confined to its physiological locations, misfolds and interacts with cellular components it does not normally encounter. The pleiotropic mechanisms of α-syn toxicity act in parallel, collectively contributing to progressive neuronal death in PD and related disorders.

### 3.5. Huntingtin

Huntington’s disease (HD) is the most common and well-studied polyglutamine neurodegenerative disease with the onset of first symptoms between the ages of 35 and 50 years. Early neurological symptoms of HD include subtle motor impairments such as clumsiness, awkward involuntary movements, bradykinesia, and rigidity. As the disease progresses, voluntary motor coordination deteriorates while involuntary movements intensify, ultimately leading to loss of mobility and communication. Death usually results from heart failure or aspiration pneumonia. Cognitive decline is also characteristic, with profound dementia in late stages [154]. The major neuropathological hallmark of HD is progressive neuronal degeneration in the striatum (caudate nucleus and putamen) and cerebral cortex, resulting from the accumulation of intranuclear inclusions (NIIs) and protein aggregates in dystrophic neurites [155,156].

Although the clinical features of HD were first described in the 19th century, its genetic basis was identified only in 1993. HD is caused by an unstable expansion of a CAG trinucleotide repeat in exon 1 of the huntingtin gene (*HTT*), leading to an elongated polyglutamine tract in the N-terminus of the protein [157]. In healthy individuals, the repeat length ranges from 6 to 35. Alleles with 27–35 repeats do not affect neurological functions but confer risk to offspring [158]. Pathogenic expansions exceeding 35 repeats cause HD, with the most severe cases containing more than 100 glutamine residues [157].

Wild-type Htt interacts with numerous proteins—234 high-confidence partners have been identified [159]—and regulates vesicle transport, cytoskeleton assembly, endocytosis, postsynaptic signaling, and transcription. It also promotes neuronal survival by enhancing expression of BDNF and NeuroD [160,161] and by inhibiting caspase-mediated apoptosis through interference with apoptosome and HIP1-HIP1-interactor protein complex formation [162,163]. Mutant Htt with an expanded polyglutamine tract disrupts these interactions, impairing neuronal function. Although mutant *HTT* is expressed throughout the body, striatal neurons—particularly medium-sized projection spiny neurons—are most vulnerable. These neurons receive dopaminergic and glutamatergic inputs from the substantia nigra, cortex, and thalamus, and produce the inhibitory transmitter γ-Aminobutyric acid (GABA). Therefore, their loss contributes to the uncontrolled movements observed in HD patients [164].

Mutant huntingtin (mHtt) is more susceptible to proteolysis than the normal protein, being cleaved by calpains, matrix metalloproteinases, and caspases [165,166,167], which generate toxic polyQ-containing fragments that further activate proteases. In both HD patients and mice, caspase-derived fragments can be detected before striatal neurodegeneration, and their production depends on the length of the polyQ tract [168,169]. In HD models, cleaved fragments of mHtt have been shown to promote cytotoxicity, excitotoxicity, and aggregate formation [170].

The intracellular aggregates of huntingtin observed in samples from HD patients are of a true amyloid nature, exhibiting a cross-β structure, as demonstrated by CR staining with green birefringence in samples from the HD frontal cortex [171] and by fibrils generated in vitro [172]. Furthermore, the antiparallel β-sheet organization of polyglutamine aggregates of huntingtin has been confirmed by numerous in vitro structural analyses [173,174,175].

Accumulation of mHtt aggregates in nuclei and neurites has been proposed to trigger neuronal death by sequestering proteins and disrupting key intracellular pathways [154]. However, inclusions may represent a protective mechanism, isolating toxic soluble N-terminal fragments or oligomers that would otherwise cause greater damage [176]. Supporting this, in vitro studies show that suppressing nuclear inclusions increases neuronal death, whereas inclusion formation reduces diffuse mHtt and enhances survival [177,178].

mHtt impairs axonal transport by altering interactions between motor proteins and microtubules, reducing delivery of neurotrophic factors such as BDNF [155,156,179]. mHtt sequesters key synaptic proteins, including Complexin II, Synaptobrevin-2, Rabphilin 3A, and PACSIN 1/Syndapin, while also disrupting neurotransmitter receptor expression and function [179,180,181]. Beyond synaptic effects, mHtt induces mitochondrial dysfunction by disrupting mitochondrial dynamics, including fission and fusion, and through interactions with key proteins such as Drp1, Mfn1/2, and OPA1. This results in altered mitochondrial morphology and defective mitophagy [182]. mHtt also reduces electron transport chain and TCA cycle activity, lowering ATP production and increasing reactive oxygen species (ROS) [182]. In addition, mHtt may activate both caspase-dependent and caspase-independent apoptotic pathways, contributing to neuronal cell death [183,184].

Thus, mutations in the *HTT* gene cause intracellular dysfunction in two ways: loss of normal wild-type huntingtin functions due to interference by the mutant protein, and a toxic gain of function resulting from altered localization and aggregation. Together, these effects disrupt multiple intracellular pathways by sequestering key components into aggregates, ultimately leading to neuronal dysfunction and death.

### 3.6. TDP-43

Amyotrophic lateral sclerosis (ALS) and frontotemporal lobar degeneration (FTLD) are two related neurodegenerative diseases that overlap clinically, morphologically, and genetically. ALS is a motor neuron disorder (MND) characterized by the premature degeneration of motor neurons in the spinal cord, brainstem, and motor cortex, leading to muscle weakness, hyperreflexia, spasticity of the limbs, and respiratory failure [185]. FTLD is a clinically and pathologically heterogeneous group of dementias, defined by degeneration of the frontal and anterior temporal lobes, resulting in progressive changes in behavior, personality, and language skills [186]. While ALS and FTLD were traditionally considered distinct disorders, they are now recognized as representing opposite ends of a spectrum of clinically, pathologically, and genetically overlapping conditions [187]. Pure forms of FTLD and ALS are connected in FTLD-MND syndromes [188].

Although most cases of ALS are sporadic, approximately 10% are familial, predominantly with autosomal dominant mutations [189]. In FTLD, roughly 30% of cases are familial [190]. Both ALS and FTLD are histopathologically characterized by abnormal accumulation of misfolded protein aggregates in affected regions of the nervous system [188]. Due to their heterogeneity, ALS and FTLD can be classified according to pathological subtypes and the presence of inclusions of specific aggregated proteins: for ALS, these include superoxide dismutase 1 (SOD1), fused in sarcoma (FUS), and optineurin (OPTN) [191]; for FTLD, tau and FUS are common. Both disorders are also associated with TAR DNA-binding protein 43 (TDP-43)-positive inclusions [186].

Direct evidence for the amyloid nature of misfolded TDP-43 aggregates in ALS-FTLD has been obtained via cryo-electron microscopy. The brain samples of patients who had ALS and FTLD with type B cortical TDP-43 pathology were characterized by the presence of round neuronal cytoplasmic inclusions of amyloid fibrils of TDP-43 in the motor cortex and spinal cord [192].

With the development of cryo-electron microscopy, growing evidence indicates that amyloid fibrils of various proteins contribute to intracellular inclusions in ALS-FTLD pathology. Amyloid filament structures of TATA-binding protein-associated factor 15 (TAF15) have been resolved from the prefrontal and temporal cortices of individuals with FTLD-FUS [13]. Additionally, it has been shown that annexin A11 (ANXA11) co-assembles with TDP-43 into heteromeric amyloid filaments in FTLD-TDP type C [14]. However, the mechanism of cytotoxicity of these amyloids has not yet been characterized. Therefore, in this review we focus on the protein TDP-43, which represents the major component of intracellular ALS-FTLD pathological inclusions.

TDP-43 is encoded by the *TARDBP* gene and belongs to the heterogenous nuclear ribonucleoprotein (hnRNPs) family, a complex and functionally diverse group of RNA-binding proteins. Under physiological conditions, TDP-43 is ubiquitously expressed and predominantly localized in the nucleus, where it regulates gene expression and multiple aspects of RNA metabolism, including transcriptional regulation, RNA trafficking and splicing, mRNA stabilization and turnover, and microRNA biogenesis [193,194,195,196]. TDP-43 is estimated to regulate more than 4000 different mRNA transcripts [197], including its own mRNA and tau transcripts [198,199]. Binding to these transcripts promotes their destabilization, leading to downregulation of tau and TDP-43 protein levels. The neurotoxicity of pathological TDP-43 is believed to arise from both a gain of toxic cytoplasmic function and a loss of physiological function associated with nuclear depletion of TDP-43. Cytoplasmic oligomers and aggregates of TDP-43 have been shown to be cytotoxic in vivo [200,201].

TDP-43 amyloid fibrils are transported into the cytoplasm and incorporated into stress granules, which leads to their irreversibility. Also, cytoplasmic amyloid conformers of TDP-43 impair axonal transport and axon growth. The nuclear depletion of TDP-43 caused by its cytoplasmic mislocalization likely produces loss-of-function effects through dysregulation of RNA metabolism, alternative splicing, and transport of dozens of transcripts [202]. TDP-43 interacts with mitochondrial outer membrane protein prohibitin 2 (PHB2) and voltage-dependent anion channel 1 (VDAC1), which are crucial receptors for Parkin-mediated mitophagy [203]. Overexpression of either wild-type or mutant TDP-43 induces mitochondrial structural and functional abnormalities, including swollen and degenerated cristae, the complete lack of cristae, reduced membrane potential, increased ROS, reduced mitochondrial ATP production, and activation of the mitochondrial unfolded protein response. Similar abnormalities have been observed in patient samples via electron microscopy. Additionally, TDP-43 pathology has been linked to disrupted Ca^2+^ homeostasis, activation of glycogen synthase kinase 3β (GSK-3β), and autophagy dysregulation [202].

## 4. Comparative Analysis of Pathological and Functional Brain Amyloids Based on Their Amino Acid Composition

**Figure 3:**
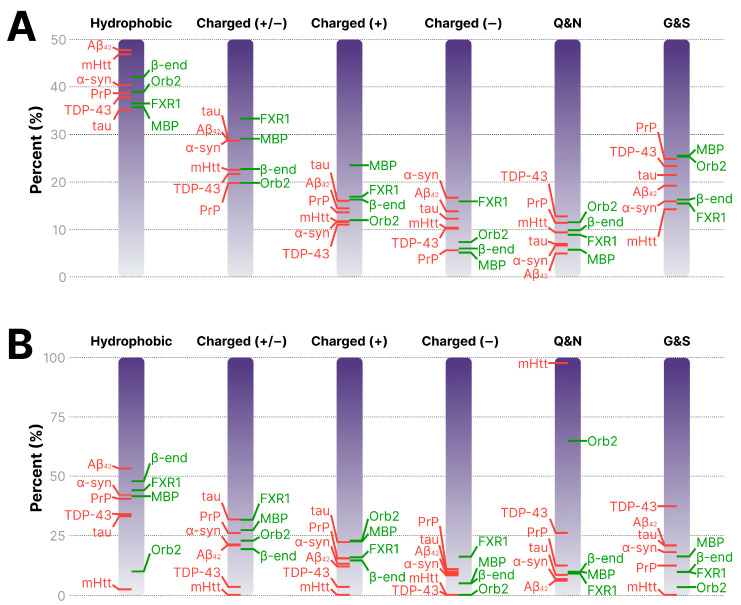
Comparative analysis of amino acid composition of pathological and functional brain amyloids. (**A**) Distribution of hydrophobic, charged (+/−), positively or negatively charged, and Q/N- and G/S-enriched residues in the full-length sequences of pathological and functional brain amyloid proteins. The percentages of amino acid residues with the corresponding characteristics are plotted along the vertical axis. (**B**) The same parameters calculated for their experimentally defined amyloidogenic core fragments. The names of pathological amyloids are marked with red lines. The names of functional amyloids are marked with green lines.

Previously, a comparative analysis of the amino acid composition of the Orb2 and Aβ40 proteins suggested that the amyloidogenic cores of pathological proteins are enriched in hydrophobic residues, whereas those of functional amyloids are predominantly hydrophilic [204]. In our opinion, drawing broad conclusions from the comparison of only two proteins seems premature. We conducted a comparative analysis of the pathological and functional brain amyloids discussed in this review (Table 1 and Table 2 and Figure 3A,B). With the exception of the fruit fly Orb2 protein, all analyzed sequences correspond to human proteins. We included β-endorphin as a representative of the large group of amyloid secretory granule proteins. The ability of β-endorphin to form amyloid fibrils has been demonstrated in at least two independent studies [3,205]. The comparison was based on several parameters such as hydrophobicity/hydrophilicity, the proportions of positively and negatively charged amino acids, and enrichment in asparagine/glutamine (Q/N) or serine/glycine (G/S) residues. The analysis was performed for both full-length proteins and their amyloidogenic core sequences, the boundaries of which were determined based on previously obtained experimental data [7,8,10,113,175,205,206,207,208,209].

The results presented in Table 1 and Table 2, as well as in Figure 3, convincingly show that the amino acid sequences of pathological and functional amyloids, as well as their amyloidogenic cores, do not differ in any of the parameters examined. For instance, the amyloidogenic core of α-syn is less hydrophobic than the cores of β-endorphin and FXR1, but more hydrophobic than those of MBP and Orb2 (Table 2 and Figure 3B). The amyloidogenic core of the mHtt protein is the most hydrophilic (Table 2 and Figure 3B), as it consists almost entirely of glutamine residues with the exception of a single phenylalanine [175]. Thus, the differences between pathological and functional amyloids are not determined by their amino acid composition, contrary to previous assumptions.

## 5. Factors Determining the Toxicity and Functionality of Amyloids

In this review, we have characterized pathological and functional brain amyloids and demonstrated that their toxicity or lack thereof cannot be explained by differences in amino acid composition. Theoretically, toxicity could be determined by the length, orientation, or location of amyloidogenic tracts within the protein. However, available experimental data contradict this hypothesis. For example, the amyloidogenic sequence is located in the N-terminal part of the mHtt protein, while in the PrP protein it is in the C-terminal part. Moreover, many pathological amyloids share a parallel in-register β-sheet structure, which is also characteristic of the functional amyloid Orb2 [8]. The length of the amyloidogenic core in the Aβ peptide is roughly similar to that of the functional amyloid Orb2. These findings challenge the assumption that toxicity is determined solely by the structural features of amyloid conformers. At the same time, the data presented in this review indicate that the presence or absence of toxicity may be determined by the localization of amyloid proteins and/or their interaction with functional partners.

### 5.1. Amyloids’ Localization

As discussed above, extracellular oligomers of PrP^Sc^ and Aβ interact with PrP^C^ on the cell surface, initiating cytotoxic cascades [76,100]. Moreover, amyloid conformers of the yeast Sup35NM protein and a synthetic amyloid peptide also bind PrP^C^ in cell models, leading to cell death [76]. These data suggest that PrP^C^ is a universal target for a wide range of extracellular amyloids, making them toxic to neuronal cells. Unlike extracellular amyloids, not all intracellular amyloids are toxic. Their subcellular localization plays a decisive role in determining whether they can access specific targets and trigger cytotoxicity. For example, all amyloid hormones in the pituitary gland are isolated from the cytosol by secretory granule membranes, preventing interaction with external targets [11]. The pH of secretory granules is lower than the intracellular pH. The acidic environment inside these granules promotes amyloid stability, but upon granule fusion with the cell membrane, the pH shift facilitates rapid fibril disassembly, releasing the hormone in its monomeric, functional form [11]. Amyloid fibrils of MBP are not isolated in membrane compartments, but are located exclusively within specialized cellular structures. As noted earlier, MBP mRNA is transported to and translated within oligodendrocyte processes. MBP molecules anchor via their N- and C-termini to the opposing membranes of these flattened processes, displacing other proteins [15]. The central fragments of the anchored MBP molecules come into close proximity and form a cross-β structure. [10]. Thus, MBP amyloid fibrils are effectively isolated within oligodendrocyte processes. Collectively, these examples indicate that the specific localization of many functional amyloids within cells prevents access to potentially dangerous targets.

### 5.2. Interaction of Amyloids with Pathological or Functional Partners

In the case of pathological amyloidogenesis, conformational changes lead not only to protein mislocalization but also to the disruption of interactions with functional partners, as well as to the appearance of interactions with new targets. All known pathological intracellular amyloids bind specific targets that trigger cell death pathways. Amyloid conformers of mHtt, α-syn, tau, and TDP-43 directly interact with mitochondrial membranes, leading to mitochondrial fragmentation and dysfunction, excessive ROS production, oxidative stress, and apoptosis [210,211,212,213].

Functional amyloid FXR1 in vertebrates and Orb2 in the fruit fly are not isolated within membrane-bound compartments or other distinct cellular structures. They are distributed throughout the cytoplasm and could theoretically interact with a wide variety of harmful targets, yet they do not exhibit toxicity. FXR1 has been shown to function as oligomers that bind numerous RNA molecules and other RNA-binding proteins [43,214]. Similarly, Orb2 forms functional oligomers that associate with RNA-binding proteins and RNA [47,48]. Apparently, specific interaction with functional partners prevents the uncontrolled growth of amyloid fibrils and sterically hinders their binding to targets that could provoke cell death. For example, expression of truncated FXR1 variants (residues 1–379 and 1–399) lacking the C-terminal RG/RGG motifs in HeLa cells drastically altered granule size and distribution of FXR1 [215]. These findings suggest that minor changes in the repertoire of FXR1’s partners strongly affect its aggregation propensity. Under physiological conditions, the complex domain architecture of FXR1 likely ensures tight regulation of its amyloidogenic potential by molecular interactors, thereby preventing cytotoxic amyloid accumulation.

### 5.3. The Concept of “Available Targets”

Based on data analyzed, we propose the “available targets” concept to explain why some amyloids are toxic while others are not. The toxicity of pathological amyloids arises from their ability to bind universal or specific targets. Interactions with key targets, such as PrP^C^ or mitochondria, initiate pathological cascades leading to cell death. Functional amyloids remain non-toxic because these targets are inaccessible to them. This inaccessibility is ensured either by localized translation and amyloids sequestration within specialized cellular structures, or by interaction with functional partners, which sterically blocks binding to harmful targets.

## 6. Conclusions

We suggest that the concept of “available targets” provides a universal explanation for the fundamental differences between toxic and functional amyloids not only in the brain. For example, the functional amyloid Pmel17 in melanocytes is stringently sequestered from the cytoplasm within melanosomes [2]. The seeds of garden pea contain amyloid aggregates of storage protein Vicilin that accumulate in vacuoles. In vitro, fibrils of this protein exhibit toxicity in mammalian cell culture. [9]. Different systemic amyloidoses are associated with the accumulation of proteins in the blood plasma, which, due to mislocalization and disrupted interactions with functional partners, form amyloid conformers and affect the cells of various organs and tissues [216,217]. Isolation of amyloids in specialized cellular compartments or their interaction with functional partners prevents amyloid toxicity. Identifying specific factors that block the interaction of amyloids with dangerous targets and limit the growth of amyloid fibrils may potentially facilitate the development of new approaches to the treatment of amyloidosis.

## Figures and Tables

**Table 1:** Comparative analysis of the amino acid composition of full-length pathological and functional brain amyloids. The table shows the percentages of hydrophobic, charged (+/−), positively or negatively charged, and Q/N- and G/S-enriched residues. The percentages were calculated using the Prot pi Tool, version 2.2.29.152 (https://www.protpi.ch/Calculator/ProteinTool, accessed on 15 September 2025).

Protein	Uniprot ID	Hydrophobic, %	Charged (+/−), %	Charged (+), %	Charged (−), %	Q/N, %	G/S, %
**Functional amyloids**							
Orb2	Q9VSR3	38.77	19.62	11.8	7.82	11.36	25.15
FXR1	P51114	36.38	33.17	16.75	16.42	8.69	15.3
MBP	P02686-3	35.54	28.94	23.35	5.59	5.58	25.38
β-endorphin	PRO_0000024975	41.94	22.58	16.13	6.45	9.68	16.13
**Pathological amyloids**							
PrP	P04156	38.53	19.48	13.42	6.06	11.25	24.67
mHtt (Q44)	P42858	46.71	22.4	11.6	10.8	9.25	14.06
TDP-43	Q13148	37.67	21.49	10.87	10.62	12.56	23.19
tau	P10636-8	34.91	28.57	15.87	12.7	6.8	21.31
α-syn	P37840	42.15	28.57	11.42	17.15	6.43	15.72
Aβ42	PRO_0000000095	47.61	28.56	14.28	14.28	4.76	19.05

**Table 2:** Comparative analysis of the amino acid composition of experimentally defined amyloidogenic core fragments of pathological and functional brain amyloids. The table shows the percentages of hydrophobic, charged (+/−), positively or negatively charged, and Q/N- and G/S-enriched residues. The percentages were calculated using the Prot pi Tool, version 2.2.29.152 (https://www.protpi.ch/Calculator/ProteinTool, accessed on 15 September 2025).

Amyloidogenic Core *	Core, % of Full Protein	Hydrophobic, %	Charged (+/−), %	Charged (+), %	Charged (−), %	Q/N, %	G/S, %
Orb2 [8]	4.4	9.68	22.58	22.58	0	64.52	3.23
FXR1 [7]	61.0	43.79	31.39	15.56	15.83	8.97	9.5
MBP [10]	32.0	41.27	26.98	22.22	4.76	9.52	15.87
β-endorphin [205]	67.7	47.6	19.05	14.29	4.76	9.52	9.52
PrP [207]	57.1	40.16	25.76	15.15	10.61	12.12	12.12
mHtt (Q44) [175]	1.4	2.22	0	0	0	97.8	0
TDP-43 [208]	21.5	33.7	3.37	3.37	0	25.85	37.08
tau [113]	16.6	32.88	31.51	21.92	9.59	8.22	20.55
α-syn [206]	44.3	41.93	20.96	12.9	8.06	6.45	17.75
Aβ42 [209]	81.0	52.93	20.58	11.76	8.82	5.88	20.59

* For each protein, a link to the article in which the amyloidogenic core was identified is provided.

## Data Availability

No new data were created or analyzed in this study. Data sharing is not applicable to this article.
